# Evaluation of Urine CCA Assays for Detection of *Schistosoma mansoni* Infection in Western Kenya

**DOI:** 10.1371/journal.pntd.0000951

**Published:** 2011-01-25

**Authors:** Hillary L. Shane, Jennifer R. Verani, Bernard Abudho, Susan P. Montgomery, Anna J. Blackstock, Pauline N. M. Mwinzi, Sara E. Butler, Diana M. S. Karanja, W. Evan Secor

**Affiliations:** 1 Division of Parasitic Diseases, Centers for Disease Control and Prevention, Atlanta, Georgia, United States of America; 2 Centre for Global Health Research, Kenya Medical Research Institute, Kisumu, Kenya; 3 Atlanta Research and Education Foundation, Decatur, Georgia, United States of America; George Washington University, United States of America

## Abstract

Although accurate assessment of the prevalence of *Schistosoma mansoni* is important for the design and evaluation of control programs, the most widely used tools for diagnosis are limited by suboptimal sensitivity, slow turn-around-time, or inability to distinguish current from former infections. Recently, two tests that detect circulating cathodic antigen (CCA) in urine of patients with schistosomiasis became commercially available. As part of a larger study on schistosomiasis prevalence in young children, we evaluated the performance and diagnostic accuracy of these tests—the carbon test strip designed for use in the laboratory and the cassette format test intended for field use. In comparison to 6 Kato-Katz exams, the carbon and cassette CCA tests had sensitivities of 88.4% and 94.2% and specificities of 70.9% and 59.4%, respectively. However, because of the known limitations of the Kato-Katz assay, we also utilized latent class analysis (LCA) incorporating the CCA, Kato-Katz, and schistosome-specific antibody results to determine their sensitivities and specificities. The laboratory-based CCA test had a sensitivity of 91.7% and a specificity of 89.4% by LCA while the cassette test had a sensitivity of 96.3% and a specificity of 74.7%. The intensity of the reaction in both urine CCA tests reflected stool egg burden and their performance was not affected by the presence of soil transmitted helminth infections. Our results suggest that urine-based assays for CCA may be valuable in screening for *S. mansoni* infections.

## Author Summary

Control efforts for schistosomiasis have in part been hampered by the lack of a sensitive and accurate test that can be utilized to rapidly map the prevalence of the disease in different areas. Recently, new tests have become commercially available that may address this problem. This study was designed to compare the new tests, which detect a schistosome antigen in patients' urine, with more traditional tests that look for parasite eggs in stool or anti-parasite antibodies in serum. We found that the new tests performed very well to detect schistosomiasis in children in western Kenya, an area with a high prevalence of *Schistosoma mansoni* infections. There was no apparent effect of soil transmitted helminth infections on the performance of the tests and the intensity of the antigen detection assays correlated well with the levels of *S. mansoni* eggs in the stool and schistosome-specific antibody in serum. Additional evaluation is needed in areas with lower schistosomiasis prevalence and intensity levels but we believe that point of contact testing of urine for schistosome antigen could be an effective tool in schistosomiasis mapping and control efforts.

## Introduction

Recently, there has been increased interest in the development and assessment of control and elimination programs for schistosomiasis [1]. For design of effective control programs, it is important to determine an accurate estimate of infection prevalence in the program area. The method most commonly used for diagnosis of *Schistosoma mansoni* infection is the detection of eggs in stool by the Kato-Katz method. Benefits of the Kato-Katz method are very high specificity, low cost, and relatively simple technologic requirements. However, the sensitivity of this method is low [2] and may be affected by day to day variability in egg excretion [3], [4], [5]. The Kato-Katz method is also time consuming and exposes laboratory workers to potentially harmful fresh stools which can contain infectious agents. In order to overcome some of the pitfalls of the Kato-Katz method, there has been interest in developing new, more sensitive tests for the diagnosis of schistosomiasis. These tests often employ immunologic methods based on the detection of antibodies or antigens in blood or urine.

Immunodiagnosis is generally more sensitive than examination of stool, particularly in low transmission areas where infection intensities are light [6]. Antibody assays can utilize crude antigen extracts such as schistosome egg antigen (SEA) or soluble adult worm antigen preparation (SWAP), or can be constructed to detect purified antigens. While methods that measure antibody levels tend to be more sensitive than Kato-Katz, parasite-specific antibodies can remain for years after the infection has been cleared. As a result, they are unable to distinguish between current and previous infections. Antibody levels in serum also do not necessarily correlate with intensity of the schistosome infection as determined by mean fecal eggs per gram.

Another method for the diagnosis of schistosomiasis is the detection of circulating anodic and cathodic antigens (CAA and CCA) in blood or urine [7]. Because CAA and CCA are released by viable adult worms, these assays are specific for current infections and can also provide some information about infection intensity [8], [9]. While CCA detection in urine can be as sensitive as a single Kato-Katz test in areas that have a high intensity of infection [10], few studies have compared the sensitivity and specificity of urine antigen detection tests with stool examination and serologic assays. This is in part because the antibodies used to detect CCA have been available in only a few laboratories and require preparation of reagents that are not readily deployable for field use. However, within the last few years, two urine CCA assays were developed and became commercially available. The first used a colloidal carbon conjugate of a monoclonal antibody specific for *Schistosoma* CCA and was designed for use in the laboratory [11]. A version of this assay was previously produced for research diagnostic purposes by European Veterinary Laboratory, and used in studies of children less than 3 years of age [12] and in school aged children with sensitivites and specificities in the low 80th percentiles when compared to stool egg data [13]. The second was a gold-conjugated, lateral flow cassette-based assay, which was designed to be a point of contact test. Since the initiation of this study, production of the test that utilized the carbon conjugate has been discontinued as a result of market considerations.

We compared the performance of the two CCA assays with that of the Kato-Katz method and a SWAP-specific IgG ELISA. This comparison is part of a larger project addressing the prevalence of schistosomiasis in pre-school and school aged children in a village in western Kenya (Verani et al., manuscript in preparation). The study site is close to the shores of Lake Victoria where our previous studies have demonstrated high levels of *S. mansoni* infection but no *S. haematobium* transmission [14], [15]. The specificities and sensitivities of the antibody and circulating antigen tests were assessed in relation to the Kato-Katz method as well as evaluated using latent class analysis (LCA), which is a method for assessing the accuracy of a test when diagnosis lacks a true gold standard [16], [17].

LCA is based on the assumption that the observed diagnostic test results for an individual are imperfect measures of the unobserved (or latent) true disease class to which this individual belongs [18]. This technique allows the prevalence of the latent classes, as well as the sensitivity and specificity of each diagnostic test, to be estimated by attributing the pattern of observed test results to the latent class membership.

Basic LCA requires the assumption of conditional independence, meaning that the diagnostic tests are assumed independent within each disease class [19]. More complex LCA methods allow modeling of the dependence structure between diagnostic tests in various ways [19], [20], [21]. Estimates from a model in which the dependence structure is incorrectly specified or even ignored could be biased [22], [23]. If a complex model is used to more accurately represent the dependence structure present in the data, however, interpretation can become difficult [23], [24] and estimation of all parameters may not be possible [20].

Statistical analysis with LCA yields more accurate sensitivities and specificities of new tests than what is obtained by comparisons to an imperfect gold standard [22], [24] and has previously been employed to evaluate tests for the diagnosis of schistosomiasis [25], [26], [27]. We also evaluated whether the results of the ELISA or CCA assays were affected by coinfection with soil-transmitted helminths (STHs) and if the results of these assays reflected the intensity of schistosome infection in the individual.

## Materials and Methods

### Ethics Statement

The study protocol was approved by the Scientific Steering Committee of the Kenya Medical Research Institute (KEMRI), the National Ethics Review Committee of KEMRI and the Institutional Review Board of the Centers for Disease Control and Prevention. Written assent and consent were obtained from study participants and their parents or guardians, respectively.

### Study Population

The samples collected in this study are part of a cross sectional study looking at childhood schistosomiasis in western Kenya. Samples were collected from children one to fifteen years of age in Usoma, a village in western Kenya on the shore of Lake Victoria. All age-eligible children were invited to participate; a total of 484 children were enrolled. Previous to this study, there had been no mass drug administration to treat schistosomiasis in this area. Any child who was determined to be schistosome or STH positive (by stool) was treated with the appropriate dose of praziquantel or albendazole, respectively.

### Sample Collection and Stool Diagnosis

Field assistants collected three stool samples on consecutive days, single mid-stream urine samples, and finger-prick quantities of blood from each child enrolled in the study. Duplicate slides of each stool sample were examined using the Kato-Katz technique. Each slide was read by two trained microscopists and any discrepancies resolved before results were recorded as eggs per gram (EPG) feces. The results for all six slides were averaged. Stools were also examined for eggs of STHs (hookworm, *Ascaris lumbricoides*, and *Trichuris trichiura*). A child was considered infected when at least one of the slides contained an egg. All data were entered into Microsoft Excel.

### IgG Detection Using SWAP ELISA

ELISA plates were coated with 100µL of 0.01mg/ml SWAP in 0.5 M sodium carbonate buffer, pH 9.6, for at least four hours at room temperature. Plates were blocked with 100µL of PBS containing 0.3% Tween 20 and 5% nonfat dried milk and were incubated overnight at 4°C. Diluted sera (1∶100 in PBS/Tween 20/.01% milk) were added to the plates and incubated at room temperature for one hour, then washed five times in PBS containing 0.05% Tween 20. Affinity purified, peroxidase labeled anti-human goat IgG (CDC) diluted 1∶1000 in PBS/Tween/0.01% milk was added for one hour at room temperature followed by five washes. Plates were developed with TMB substrate (Kirkegaard & Perry Laboratories, Gaithersburg, MD) and stopped with 18% sulfuric acid. The plates were read on a Molecular Diagnostics Vmax microplate reader (Molecular Devices Corporation, Sunnyvale, CA) at 450 nm and analyzed with Softmax software (Molecular Devices). To ensure consistency between plates a standard curve was developed and included on each plate. A 1∶3 serial dilution curve was made from highly positive serum from adult male car washers from the area and assigned arbitrary units. A four-parameter curve fitting model was used to assign units to each unknown sera. The positive cutoff value was set at two standard deviations above the average anti-SWAP IgG value of 13 different ‘normal human serum’ samples (sera from non-endemic individuals) run in conjunction with the patient samples.

### CCA Assays

Both CCA urine assays were obtained from Rapid Medical Diagnostics (Pretoria, South Africa) and performed at ambient temperature according to the manufacturer's instructions. The CCA urine assays were not available at time of sample collection; therefore, collected urine was stored at −20°C until the assays were run. Urine samples were completely thawed and vortexed before use. Briefly, for the laboratory-based test, 25 µL of urine was added to a tube containing dried carbon conjugated antibody, along with 75 µL of buffer that was supplied by the company. Test strips were added, and allowed to develop for 40 minutes. Strips were removed, allowed to dry, and read against standards provided by the manufacturer. As the CCA strips were read, they were compared to the standards and scored as 0, +, ++, or +++. A score of 0 indicated a negative result, + indicated that a band was as dark as the 100 standard, ++ indicated a band was as dark as the 1,000 standard, and +++ indicated that a band was as dark as the 10,000 standard. Results were determined in a blinded fashion by at least two individuals. For the cassette assay, one drop of urine was added to the well of the testing cassette and allowed to absorb. Once fully absorbed, one drop of buffer (provided with the kit) was added to the well and the assay was allowed to develop. Twenty minutes after the buffer was added the tests were read. Tests were considered invalid if the control bands did not develop, or if the test sat for 25 minutes after the buffer was added before being read. In these cases, the sample was rerun with a new test cassette. Results were determined in a blinded fashion by at least two individuals and scored as either negative, +, ++, or +++. Due to the lack of standards designed for this test, the classification of the positive results as weak or strong was more subjective than the laboratory-based carbon assay.

### Statistical Analysis

Sensitivities and specificities were first estimated using the Kato-Katz method as the reference test. Given that the Kato-Katz method yielded a positive result, the sensitivity of a test was defined as the percentage of subjects with a positive test result. Similarly, those with a negative Kato-Katz result were used to find the specificity of each test by finding the percentage of subjects with a negative result.

We next performed latent class analysis (LCA), in which the results of the four diagnostic assays (Kato-Katz, anti-SWAP IgG ELISA, and the two urine CCA assays) were combined as indicators of an underlying latent class, the true infection status of the individuals (S. *mansoni* positive or negative). Using the relationship between the true disease class and the observed test patterns, sensitivity and specificity were estimated for each test. While the assumption that all tests are conditionally independent given the true disease status [23] is often made in LCA, it was not made in this analysis because the two CCA diagnostic tests identify the presence of the same antigen in urine samples and are expected to be correlated. The addition of a latent variable allowed for conditional dependence between these two tests [21]. LCA was performed using the software BLCM: Bayes Latent Class Models version 1.3 [28].

For evaluations of the effect of STH infection on assay performance, statistical analyses were performed using Kruskal-Wallis nonparametric one-way analysis of variance (ANOVA) and Fisher's exact test for contingency analysis. Similarly, analyses of test results in relation to schistosome infection intensity were performed by Kruskal-Wallis ANOVA. These analyses were performed using InStat GraphPad Software version 3.05 (GraphPad Software, LaJolla, CA).

## Results

### Prevalence and Intensity of *S. mansoni*

Between September and December 2007, 247 boys and 237 girls between the ages of 1 and 15 provided stool, urine and blood for diagnostic testing. Of the 484 children enrolled in this study all three stool samples were obtained from 482 individuals. Of these, 187 (38.8%) tested positive for *S. mansoni* by the Kato-Katz method. Among the positive individuals, 87 (46.5%) were classified as having a low intensity infection (0–100 EPG), 68 (36.4%) had moderate infections (101–400 EPG), and 32 (17.1%) had heavy infections (>400 EPG). Nineteen (3.9%), 77 (15.9%) and 157 (32.4%) individuals were infected with *Ascaris lumbricoides*, hookworm, and *Trichuris trichiura*, respectively.

**Figure 1:**
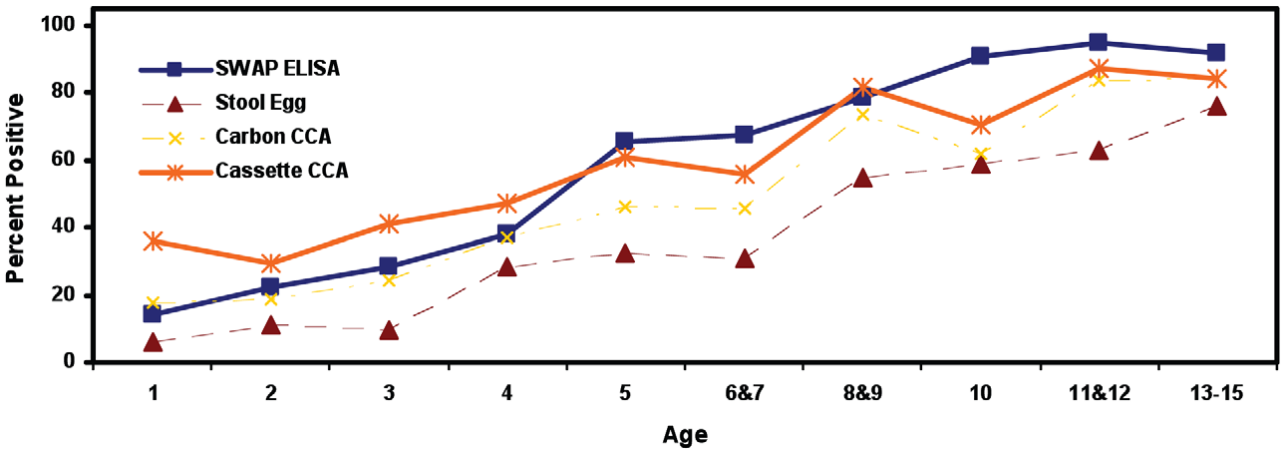
Prevalence of schistosome positive individuals by age. Prevalence increases with age for all 4 assays.

The number of children positive by each test is shown in Table 1. Of the 484 individual sera evaluated by the SWAP ELISA, 298 (61.6%) were positive for *S. mansoni* antibodies. Of the 426 patients' urines tested for CCA by the laboratory-based carbon assay, 226 (53.1%) were positive; 423 urine samples were tested for CCA by the cassette assay and 264 (62.4%) individuals were positive. An increase in prevalence with age was observed for all 4 tests (Figure 1).

**Table 1:** Prevalence of S. *mansoni* according to each diagnostic method.

Test Method	# tested	# positive	% positive (CI†)
Kato-Katz	482	187	38.8 (34.4, 43.3)
SWAP ELISA	484	298	61.6 (57.1, 65.9)
Carbon CCA	426	226	53.1 (48.2, 57.9)
Cassette CCA	423	264	62.4 (57.6, 67.0)

**†:** Exact 95% confidence interval.

A complete specimen set (three consecutive stools, blood, and urine) was available for 413 of the 484 children enrolled in the study so that all four tests could be performed. Of these, 143 (34.6%) were *S. mansoni* positive by all the tests and 95 individuals (23.0%) were negative for *S. mansoni* in all assays. A total of 53 individuals (12.8%) were egg negative, but positive by both CCA assays and the SWAP ELISA. Forty-four individuals (10.7%) were positive only by the SWAP ELISA, 23 (5.6%) were positive only by the cassette CCA assay, and three (0.7%) were positive only by the carbon CCA assay. Four individuals (0.9%) were positive by the Kato-Katz method and not by any other test; three of these children had relatively light infections (96, 102, and 18 EPG) and one had an EPG count of 1,382. The remaining 48 children (11.6%) were positive by either two or three of the testing methods. There were 70 individuals who were carbon CCA positive, but egg negative. Out of these individuals, 56 (80%) were positive by the SWAP ELISA. There were 99 individuals who were cassette CCA positive but were egg negative. Out of these individuals, 65 (65.6%) were positive by the SWAP ELISA.

### Sensitivities and Specificities of the Diagnostic Assays

We analyzed the sensitivity and specificity data in two ways (Table 2). In the first analysis, we compared the results to the detection of eggs in stool with the Kato-Katz method, an imperfect standard. When using the Kato-Katz results to determine the sensitivities and specificities of the CCA urine assays, the sensitivities were high, 88.4% and 94.2% for the carbon CCA assay and the cassette CCA assay, respectively. However, by this analysis, the specificities of the CCA assays were low, 70.9% for the carbon assay, and 59.4% for the cassette assay. The SWAP ELISA had a sensitivity of 92% but a specificity of only 57.3% when using the Kato-Katz method as the reference.

**Table 2:** Sensitivity and specificities of tests in comparison to Kato-Katz and using LCA.

Kato-Katz as Gold Standard		
Test Method	Sensitivity, % (CI†)	Specificity, % (CI†)
Kato-Katz	N/A	N/A
SWAP ELISA	92.0 (87.1, 95.4)	57.3 (51.4, 63.0)
Carbon CCA	88.4 (82.6, 92.8)	70.9 (64.9, 76.4)
Cassette CCA	94.2 (89.6, 97.2)	59.4 (53.0, 65.5)

Latent Class Analysis		
Test Method	Sensitivity, % (CI‡)	Specificity, % (CI‡)
Kato-Katz	74.1 (67.0, 80.8)	95.3 (90.9, 98.3)
SWAP ELISA	96.3 (92.2, 98.9)	68.7 (61.2, 75.8)
Carbon CCA	91.7 (86.3, 95.9)	89.4 (83.1, 94.7)
Cassette CCA	96.3 (92.0, 99.0)	74.7 (67.0, 81.6)

†Exact 95% confidence interval.

‡95% credible interval.

We also analyzed the data using LCA to establish the infection status of the study participants (Table 2). In this analysis, the Kato-Katz method had the lowest sensitivity (74.1%) out of the diagnostic tests. Both the SWAP ELISA (96.3%) and the urine CCA assays (91.7% and 96.3%) had high sensitivity by LCA. The specificities for these assays were not as high as the Kato-Katz method, but were higher than the values obtained when using Kato-Katz as the gold standard.

### Effects of STH Infections and Intensity of Infection on Assay Performance

**Figure 2:**
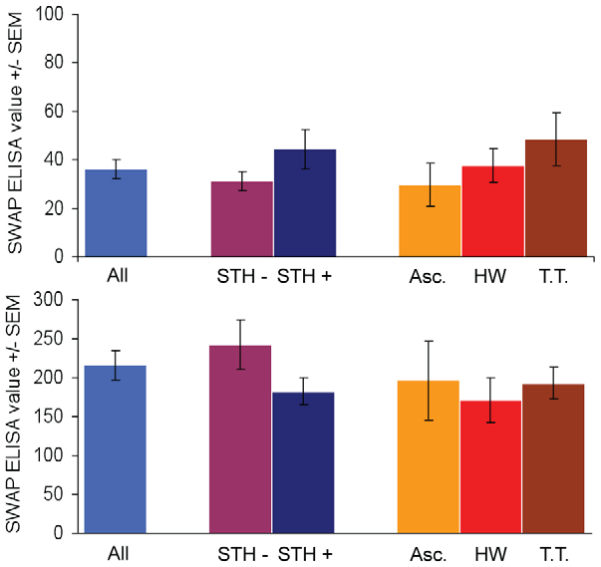
STH infection status does not affect SWAP-ELISA values. Comparison of SWAP ELISA values in schistosome negative (top) and schistosome infected (bottom) individuals based on STH infection status. Y-axis scales differ in order to better analyze the schistosome negative individuals. There are no significant difference between SWAP ELISA values based on STH infection status (p = 0.4263 and 0.8868, respectively) with one-way analysis of variance (Kruskal-Wallis ANOVA).

To determine if infection with *Ascaris lumbricoides*, hookworm, or *Trichuris trichiura* modified the responses in the SWAP ELISA, we evaluated serologic responses of individuals that either were, or were not infected with STHs. There were no significant differences in the SWAP ELISA values between groups that did or did not have STHs for either schistosome negative or positive individuals (Figure 2). For this analysis, individuals were classified as ‘schistosome negative’ only when they were negative by all diagnostic assays. Individuals were classified as ‘schistosome positive’ when they were positive by the Kato-Katz method or either of the urine CCA assays. Similarly, we compared the CCA results from people infected with STHs to individuals who were negative for STH eggs by the Kato-Katz method (Table 3). The percentages of CCA positive individuals did not significantly change based on STH infection status of the individuals, suggesting that STH infections do not influence the urine CCA assay results.

**Table 3:** Urine CCA assays are not affected by STH infection status.

	CCA+	Carbon CCA+	Cassette CCA+	CCA−	ELISA+
*S. mansoni* egg negative, all	112/261 (42.9%)	74/254 (29.1%)	102/251 (40.6%)	149/261 (57.1%)	119/261 (45.6%)
*S. mansoni* egg negative, STH+	43/102 (42.2%)	30/100 (30.0%)	41/99 (41.4%)	59/102 (57.8%)	47/102 (46.1%)
*S. mansoni* egg negative, STH−	69/159 (43.4%)	44/154 (28.6%)	61/152 (40.1%)	90/159 (56.6%)	72/159 (45.3%)

**Figure 3:**
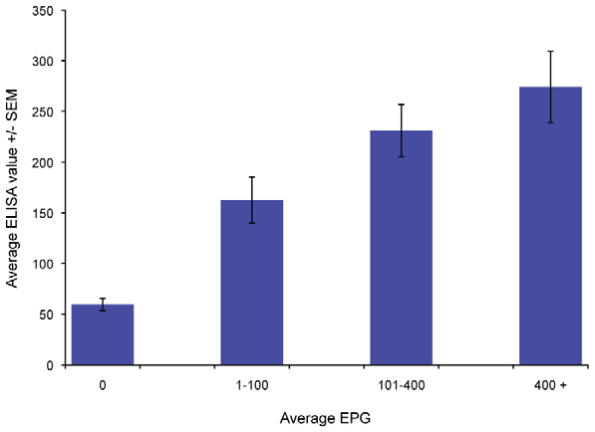
SWAP-ELISA values correlate with level of schistosome infection. Average SWAP-ELISA values for the negative, light, moderate and heavy intensities of infection as determined by average EPG (p<0.001, Kruskal-Wallis ANOVA).

**Figure 4:**
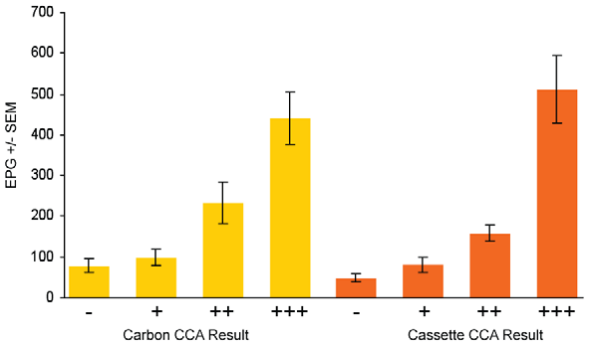
CCA assay band intensity correlates with level of schistosome infection. The intensity of the results of the CCA assays are associated with schistosome infection intensity (p<0.0001, Kruskal-Wallis ANOVA), as determined by the Kato-Katz method (EPG).

We also tested whether the ELISA and CCA results reflected the intensity of infection in the individuals as measured by stool egg count. SWAP ELISA values were averaged for the individuals who had varying levels of intensity: not infected (no eggs found), light infection (0–100 EPG), moderate infection (101–400 EPG) or heavy infections (>400 EPG). The levels of total anti-SWAP IgG were correlated with intensity of infection as determined by EPG (p<0.0001, Figure 3). To determine if band intensity of the CCA assays correlated with intensity of infection, we compared the average EPG found in each of the individuals to band strength (Figure 4). For both CCA tests, band intensity was associated with intensity of infection (p<0.0001).

## Discussion

The Kato-Katz assay has long been the backbone of schistosomiasis diagnosis in endemic areas. However, even when multiple samples are tested, the Kato-Katz method has inadequate sensitivity, especially in areas with lower rates of transmission [29]. This results in the challenging task of trying to evaluate other tests in comparison to an imperfect ‘gold standard’. When using the Kato-Katz as the ‘gold standard’ to determine the sensitivities and specificities of the CCA urine assays, the sensitivities are rather high but the specificities are quite low. The low specificities obtained when using Kato-Katz as the ‘gold standard’ may be explained by the low sensitivity of the Kato-Katz method. Thus, to more accurately assess the sensitivities and specificities of these assays, we analyzed the results using LCA. We chose the Bayesian approach to Latent class analysis, considering the two CCA diagnostic assays correlated because they test for the same antigen in the same sample (urine). Sensitivities and specificities of each of the four tests were calculated based on the latent variable ‘true disease status’.

As expected, the Kato-Katz method had the lowest sensitivity when analyzed by LCA. The SWAP ELISA and the cassette urine CCA assay were the most sensitive assays, albeit also the least specific. It is possible that false positive antibody responses could be present in children who were exposed to schistosome antigens but not infected (e.g., in utero, during the pregnancy of an infected mother) or in older children who had been infected then cleared the infection due to natural worm death. Schistosomes that typically infect wild mammals, including some new and hybrid species, have also been described in the Lake Vitoria Basin near Kisumu [30]. However, it is not known how human exposure to these other schistosomes may affect performance of the ELISA.

False positive CCA results may occur if the individual being tested has haematuria or pio-uria due to urinary tract infection, as stated on the CCA assay technical brochure. However due to the age of these children we feel that this would not likely make a large impact on our study. Similarly, we do not know what the effect of having frozen the urine had on the assay performance as we did not test any urine that had not been frozen. The product insert states that urine can be frozen for up to a year but we are unable to comment on whether or not the frozen urine was any less sensitive than urine collected and tested immediately.

Of the 53 individuals who were negative for eggs in stool but positive by both of the CCA assays and the SWAP ELISA it is likely that they had light infections that were missed by the Kato-Katz even though stools were collected on three consecutive days. Of the four children who were positive by stool examination and not by any other test, three children had lighter infections (96, 102, and 18 EPG) that may have been missed by the other diagnostic tests. However, one of the children had a high intensity infection (1,382 EPG), which was unlikely to be missed by the other assays. It is possible that the stool samples were mislabeled and not from the same individual that provided the other two samples types. This highlights a limitation of stool and urine data in that sample collection is typically not directly observed and either urine or stool may be substituted with samples from other individuals. In contrast, this is usually not a concern for blood samples collected by study personnel.

Other factors that may have contributed to incongruous results between the various assays include a poor understanding of whether CCA levels demonstrate diurnal variations. In addition, volume of fluid intake or number of previous urinations in a given day may affect urine CCA concentration. Also, while our assumption was that most of the children in this study had not previously been treated for schistosomiasis as no mass treatments had recently been performed in this area, it is possible that some families had migrated into the area and that children may have been treated elsewhere and could thus be antibody positive but egg and antigen negative. Finally, although HIV-1 prevalence levels are high in this area of western Kenya and could theoretically affect antibody responsiveness, we do not expect that HIV-1 coinfection would significantly affect our observed results as the age of the majority of participants was such that previous exposure to HIV-1 was unlikely.

In addition to the accuracy of a screening test, it is important to also consider practicality when comparing different tests [31]. Although the Kato-Katz test can be done at a relatively low cost, there are other factors that make this method less than ideal. For example, the Kato-Katz method requires one or more individuals trained in stool sample preparation and microscopy. There are also significant expenses associated with transport costs and the field staff time necessary for multiple field trips, to obtain consecutive stool samples and to provide treatment. In contrast, the cassette CCA assay is designed for point of contact use. Thus, health workers could combine screening and treatment during a single trip which would save on costs and possibly reduce the number of missed treatments of individuals who cannot be found on a follow-up visit. Currently, the individual test cost of the CCA assays is relatively high ($1.98 US per test) but future comparisons of expenses should include personnel time, transport and equipment expenses to determine which approach is more cost effective. In addition, cost of the CCA test may be reduced with greater use and increased scale of production.

Although the SWAP ELISA had similar sensitivity as the cassette CCA assay, this assay is less practical in field based control programs because of the requirements for reagents and equipment, as well as the need for specialized training of laboratory personnel. Also, the inability of the total IgG antibody test to distinguish between past and current infections makes this a poor test for assessing prevalence in populations that have already received treatment.

Our results are consistent with those of a recently published study that found good agreement between urine CCA assay results and a single stool analyzed by the Kato-Katz method [32]. This study evaluated a total 171 children, ages 6 to 17, in 11 schools along the Kenyan and Tanzanian shore of Lake Victoria, including 14 children from Usoma, the school that serves the children in our study. Both studies found a strong correlation between stool egg concentration and intensity of the CCA test band ([32] and Figure 4).

Like the authors of this previous study [32], we conclude that the CCA urine assays are an effective screening tool for *S. mansoni* infections in areas of high prevalence. The CCA urine assays were more sensitive than examination of three stools by Kato-Katz and were as sensitive as the adult worm-specific antibody tests. The urine CCA assays are also easy to use and less time consuming than the other methods currently employed for *S. mansoni* screening. CCA assays also have the potential to asses cure, as CCA should not be present after the resolution of infection [33]. However, in this study, we were not able to assess cure rates or the ability of the tests to detect resolution of infections as we did not collect additional samples after treatment. Future studies including urine CCA assays should address this question as well as evaluating the performance of the tests in areas with lower intensities of infection. Table 4 summarizes the strengths and weaknesses of the assays used in this study for population screening purposes. How data from these different assays can be used to inform treatment decisions at the local level has been discussed by Standley et al. in a recent publication [34].

**Table 4:** Comparison of characteristics important for effective screening test.

	Kato-Katz	SWAP ELISA	Carbon CCA	Cassette CCA
**Specimens**				
Ease of collection	+	++	+++	+++
Specimen of use	Stool	Blood	Urine	Urine
**Assay Performance**				
Ease of Use	++	+	+++	+++
Speed of Result	+	+	++	+++
**Relevance of Field Use**				
Commercial Availability	+++	+	−	+++
Test Supply Costs (Low)	+++	++	N/A	+
Indirect Test Costs (Low)	+	++		+++

Results from this study indicate that the urine CCA assays are at least as sensitive as the Kato-Katz method of testing for schistosome eggs in stool. The company that produced both CCA tests has now halted production of the carbon CCA assay. Results from the discontinued test are included here because results from that assay contributed to the study analysis. Production of the cassette CCA assay continues and its ease of use and relatively simple sample collection make it an attractive tool for screening for *S. mansoni* infections in control programs.

## Supporting Information

Checklist S1 STARD checklist.
(0.05 MB DOC)

Flowchart S1 STARD flowchart.
(0.04 MB DOC)
