# Supplementary material for: Evaluation of Urine CCA Assays for Detection of Schistosoma mansoni Infection in Western Kenya
Source: PLoS Negl Trop Dis. 2011 Jan 25;5(1):e951. doi: 10.1371/journal.pntd.0000951 (PMC3026766; doi:10.1371/journal.pntd.0000951)
Supplement: Flowchart S1 — STARD flowchart. (0.04 MB DOC) [file pntd.0000951.s002.doc]

Eligible patients

n = 484

Excluded patients

n =61

Reasons: inadequate volume of urine or blurred number on tube

Tested by cassette CCA

n = 423

Result missing for one of other assays

n = 10

Complete specimen set

N = 413

|  | Kato Katz –  ELISA –  Carbon CCA – | | Kato Katz –  ELISA –  Carbon CCA + | | Kato Katz +  ELISA –  Carbon CCA – | | Kato Katz +  ELISA –  Carbon CCA + | | Kato Katz –  ELISA +  Carbon CCA – | | Kato Katz –  ELISA +  Carbon CCA + | | Kato Katz +  ELISA +  Carbon CCA – | | Kato Katz +  ELISA +  Carbon CCA + | |
| --- | --- | --- | --- | --- | --- | --- | --- | --- | --- | --- | --- | --- | --- | --- | --- | --- |
| Cassette CCA | - | + | - | + | - | + | - | + | - | + | - | + | - | + | - | + |
|  | 95 | 23 | 3 | 11 | 4 | 2 | 0 | 5 | 44 | 12 | 3 | 53 | 5 | 9 | 1 | 143 |
| LCA +  Probability | 0.00 | 0.00 | 0.01 | 0.16 | 0.03 | 0.22 | 0.25 | 0.92 | 0.03 | 0.22 | 0.25 | 0.92 | 0.64 | 0.94 | 0.94 | 1.00 |
